# Developing accurate prediction systems for the terrestrial environment

**DOI:** 10.1186/s12915-018-0515-6

**Published:** 2018-04-18

**Authors:** David B. Lindenmayer

**Affiliations:** Fenner School of Environment and Society, The Australian National University, Canberra, ACT 2601 Australia

## Abstract

In recent decades, meteorologists have made remarkable progress in predicting the weather, thereby saving lives and considerable sums of money. However, we are way behind when it comes to predicting the effects of environmental change on ecosystems, even when we are ourselves the agent of such change. Given the substantial environmental problems facing our living planet, and the need to tackle these in an ecologically responsible and cost-effective way, we should aspire to develop terrestrial environmental prediction systems that reach the levels of accuracy and precision which characterize weather prediction systems. I argue here that well designed, long-term monitoring programs will be key to developing robust environmental prediction systems.

Environmental prediction in terrestrial ecosystems is a tough challenge. No two parts of a landscape are the same and many factors drive, for example, the distribution and abundance of key elements of the biota and how they respond to a changing environment, especially changes resulting from human disturbance. Almost 60 years ago, Eugene Odum argued that because aquatic, marine and atmospheric environments are dominated by physical processes, it is far easier to develop prediction systems for them than for terrestrial ecosystems, which are dominated by biological processes [1]. Nevertheless, there is increasing public expectation that the large sums of money spent on the environment will deliver the desired objectives, and this necessitates being able to accurately forecast what will happen in the future - for example what will occur when particular kinds of land management are adopted in projects aimed at achieving the widespread landscape restoration of formerly degraded forested land.

Of the many things needed to improve our ability to develop more accurate prediction systems for the terrestrial environment, I argue that one of the most important is the establishment and maintenance of well-designed long-term ecological monitoring and research programs. These are essential to gather the kinds of data needed to develop robust environmental prediction systems [2]: it is not possible to make robust predictions of the future without an understanding of what has happened in the past (Fig. 1). Approaches such as systematic reviews and meta-analyses are extremely valuable for synthesizing bodies of knowledge to highlight evidence for particular kinds of responses to environmental change. For example, a global meta-analysis of multiple forest restoration studies has provided insights into the ecological drivers of restoration success [3]. However, these methods are dependent on combining many different datasets and are complementary to, but not a substitute for, well targeted long-term environmental monitoring programs. The most valuable systematic reviews and meta-analyses are underpinned by robust empirical, field-based studies, including investigations based on long-term environmental monitoring programs [2].

**Fig. 1:**
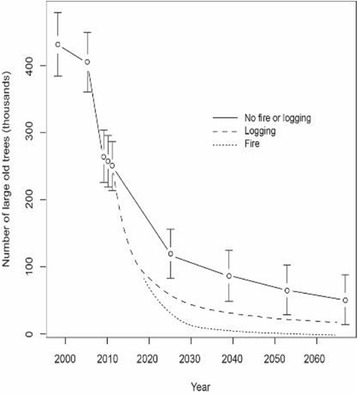
Long-term data showing the decline in the abundance of large old trees since 1997 in the Mountain Ash forests of south-eastern Australia. The figure shows temporal changes in abundance based on field-based re-measurements of trees to 2011. Projected tree abundance after 2011 is based on 14-year time steps in a Markov-chain model for transition probabilities of trees based on their deterioration in condition and eventual collapse. Projections are made to 2067 when new cohorts of large old trees will first start to develop in Mountain Ash forests. The diagram also shows different projections for the future abundance of large old trees in response to additional fire and ongoing logging in the Mountain Ash forest (see [9] for further details)

A key consideration is the design of monitoring and data collection programs. I have been involved in a recent (currently unpublished) study of population trends in Australian birds which found that, for an array of reasons, over 90% of datasets collected by agencies and volunteers were unusable. In fact, their inherent problems would likely make prediction worse, not better, because of the massive amount of “noise” which can obscure key signals in long-term species population trends. The lesson here then is that more data is not better data and big data is not a substitute for quality data.

There are many examples demonstrating the value of long-term monitoring and research in helping to develop environmental prediction systems [2]. One with which I have had a close involvement is in the tall wet Mountain Ash (*Eucalyptus regnans*) eucalypt forests of south-eastern Australia, an ecosystem currently dominated (to the extent of 98.8%) by regrowth forest (Fig. 2). Large, old, hollow trees are a key structural feature of these native forest ecosystems because they provide critical habitat for biodiversity, store large amounts of carbon, influence the water cycle, and have profound effects on fire dynamics. Repeated surveys over a 20-year period have described temporal changes in the condition of the remaining large old trees and shown that their numbers have almost halved since 1997 (Fig. 1). These data facilitate future projections of the tree population over the next 50 years and it is predicted that by the middle of this century, only one-seventh of the original population will remain (Fig. 1). The reasons for such a major decline, both past and projected, include extensive and intensive historical and current logging operations, repeated past fires (followed by post-fire salvage logging), and the prolonged period (> 120 years) it takes to grow a large old tree. Long-term data on biodiversity in the Mountain Ash ecosystem show that the decline in populations of many species of cavity-dependent arboreal marsupials closely mirrors that of the decline in populations of large old trees. These long-term datasets therefore provide a powerful system for predicting the dynamics not only of keystone ecosystem structures (i.e., large old trees), but also the array of faunal species associated with them, such as the critically endangered Leadbeater’s Possum (*Gymnobelideus leadbeateri*), and the vulnerable Greater Glider (*Petauroides volans*) and Yellow-bellied Glider (*Petaurus australis*). This prediction system has, in turn, provided land managers with the opportunity to rethink and revise the kinds of policies and management practices that are needed to better conserve both trees and associated cavity-dependent fauna [4]. This can be codified as projections with different trajectories corresponding to different future management options (Fig. 1), akin to economic forecasting under different policy settings. Such forecasts can assist policy makers and resource managers in making informed decisions. In the particular case of the wet forests of Victoria, the environmental predictions highlight a need to protect all existing large old trees in an attempt to prolong their standing life, and the benefits of a major reduction in logging to allow new cohorts of trees to grow through to an old growth stage.

**Fig. 2:**
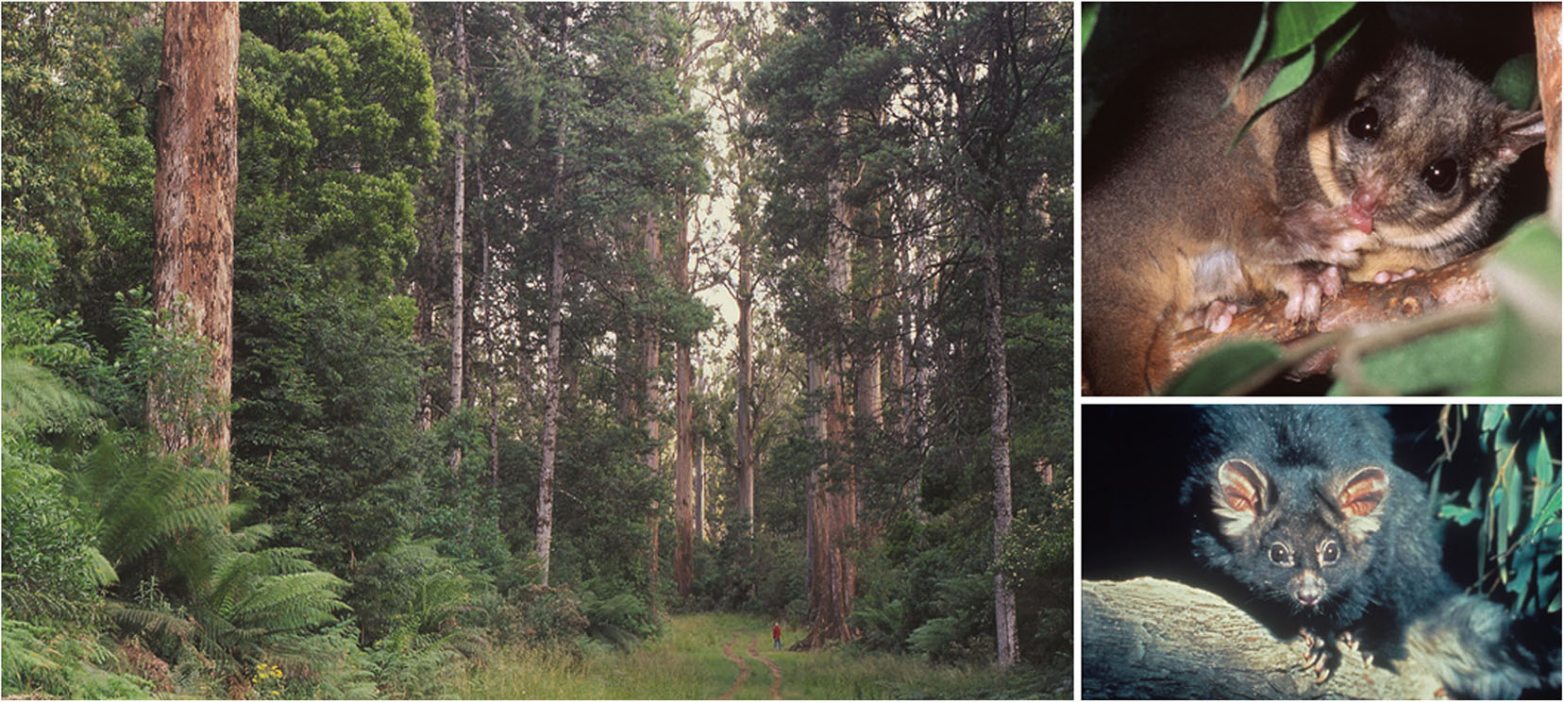
Stand of large old Mountain Ash trees (photo by Esther Beaton). Top right. Leadbeater’s Possum (photo by David Lindenmayer). Bottom right. Greater Glider (photo by David Lindenmayer)

Of course, there are always uncertainties in any predictions, including those shown in Fig. 1, although the relatively tight prediction intervals in projected changes in tree populations (in the absence of further fire or ongoing logging) suggest that alternative trajectories to the catastrophic future declines are highly unlikely. In fact, while there will likely always be ecological surprises in many ecosystems worldwide, they are far more likely to be detected early (when there are opportunities to do something about them) in those environments that have been subject to rigorously designed long-term monitoring and research [5]. In other cases, projections may be inaccurate, but these too offer an opportunity for learning and identifying ways to make better predictions in the future. Many scientists consider that more and better lessons are learned from failures rather than successes in ecological and environmental science [6].

The value of long-term monitoring and research has been established in numerous studies over the past 30 years. For example, long-term studies tend to be more highly cited than shorter investigations [7]. Despite the exceptional value of long-term work, monitoring is widely regarded as a “Cinderella” science—it is largely overlooked, the last thing funded, and the first thing cut when budgets are tight [2]. Are there ways to secure long-term monitoring and research? One key way is for researchers to highlight that the data they are gathering from long-term monitoring and research have value for environmental prediction, as in the case of the example in Fig. 1. Another is to re-use the data in other practical and applied ways. For example, the data that underpin Fig. 1, as well as data from other allied monitoring programs in the Mountain Ash ecosystem, were used to develop economic and environmental accounts that highlight the economic value of a suite of natural assets, including water, carbon, timber, tourism, and biodiversity [8]. Such accounts are best populated with robust long-term monitoring data. The key point is that demonstrating the manifold practical uses of monitoring will more likely help convince funders that it is important to maintain ongoing financial support for these programs.

Significant improvements in prediction systems will require far more terrestrial environments to be the target of robust, well designed, and long-persisting long-term monitoring and research programs. One way to do this is to ensure that monitoring is a fundamental part of all major environmental programs. Many past programs around the world, including those in which billions of dollars have been spent, have not been monitored or monitored very poorly (ranging from river and land restoration programs to agri-environment schemes [2]). Vitally important data have not been gathered and important insights and opportunities have been lost for environmental learning and for building environmental prediction systems. Demanding that all major environmental programs include robust, well designed long-term monitoring would provide the opportunity to gather the data that are fundamentally important to developing the environmental prediction systems so desperately needed for better tackling the numerous problems facing the world’s terrestrial ecosystems.
